# Cost-effectiveness analysis for a tele-based health coaching program for chronic disease in primary care

**DOI:** 10.1186/s12913-017-2088-4

**Published:** 2017-02-15

**Authors:** Erja Oksman, Miika Linna, Iiris Hörhammer, Johanna Lammintakanen, Martti Talja

**Affiliations:** 1 Päijät-Häme Social and Health Care District, Keskussairaalankatu 7, 15 850 Lahti, Finland; 2 Healthcare Engineering, Management and Architecture Institute, Aalto University, Espoo, Finland; 3 Department of Social and Health Management, University of Eastern Finland, Joensuu, Finland

**Keywords:** Health coaching, Self-management, Cost-effectiveness, Chronic disease, 15D, Health-related quality of life

## Abstract

**Background:**

The burden of chronic disease and multimorbidity is rapidly increasing. Self-management support interventions are effective in reduce cost, especially when targeted at a single disease group; however, economical evidence of such complex interventions remains scarce. The objective of this study was to evaluate a cost-effectiveness analysis of a tele-based health-coaching intervention among patients with type 2 diabetes (T2D), coronary artery disease (CAD) and congestive heart failure (CHF).

**Methods:**

A total of 1570 patients were blindly randomized to intervention (*n* = 970) and control (*n* = 470) groups. The intervention group received monthly individual health coaching by telephone from a specially trained nurse for 12-months in addition to routine social and healthcare. Patients in the control group received routine social and health care. Quality of life was assessed at the beginning of the intervention and follow-up measurements were made after 12 months health coaching. The cost included all direct health-care costs supplemented with home care and nursing home-care costs in social care. Utility was based on a Health Related Quality of Life (HRQoL) measurement (15D instrument), and cost effectiveness was assessed using incremental cost-effectiveness ratios (ICERs).

**Results:**

The cost-effectiveness of health coaching was highest in the T2D group (ICER €20,000 per Quality-Adjusted Life Years [QALY]). The ICER for the CAD group was more modest (€40,278 per QALY), and in the CHF group, costs increased with no marked effect on QoL. Probabilistic sensitivity analysis indicated that at the societal willingness to pay threshold of €50,000 per QALY, the probability of health coaching being cost effective was 55% in the whole study group.

**Conclusions:**

The cost effectiveness of health coaching may vary substantially across patient groups, and thus interventions should be targeted at selected subgroups of chronically ill. Based on the results of this study, health coaching improved the QoL of T2D and CAD patients with moderate costs. However, the results are grounded on a short follow-up period, and more evidence is needed to evaluate the long-term outcomes of health-coaching programs.

**Trial registration:**

NCT00552903 [Prospectively registered, registration date 1^st^ November 2007, last updated 3^rd^ February 2009].

## Background

In the European Union (EU), approximately 50 million people live with multiple chronic diseases, and this is one of the leading causes of growing healthcare costs. It is estimated that chronic diseases inflict 70–80% of total healthcare costs in EU countries [1]. In Finland (pop. 5.4 million in 2011), 37.2% of the population had at least one chronic disease or health problem in 2011 [2]. Therefore, how to manage the burden of chronic disease is a key question for policy makers.

Self-management support interventions are widely recognized as a promising approach to enhance health outcomes and contain costs in chronic care. Previous studies suggest that self-management interventions improve clinical outcomes, self-efficacy, quality of life and self-management behaviour [3–5]. They have also been successful in reducing hospitalization and healthcare costs, especially when the intervention has been focused on a single disease. The most promising results have been observed in respiratory and cardiovascular diseases [6].

However, the economic evaluation (cost effectiveness and cost-utility analysis) of self-management interventions is still scarce, although cost-effectiveness analysis has become a standard practice in evaluating, e.g. medical treatments [7–10]. This may be due to methodological challenges; self-management interventions are often complex interventions, and the standard experimental setting, a randomized controlled trial (RCT), is difficult to put into practice in real life [11, 12]. Furthermore, routinely collected administrative and clinical data typically lack the important measurements needed in the assessment of self-management interventions [13].

Health coaching is patient-oriented health promotion and education within a coaching context that emerged from the motivational interviewing concept [3]. The purpose of health coaching, as defined by Palmer et al. [14] is to motivate the patient to achieve a goal that enhances quality of life and improves health. A coach’s role is to help patients weigh options, make choices and plan and identify challenges to help them change for the better [14].

Telephone-based health-coaching intervention was launched in November 2007 in the Päijät-Häme area in Finland. The number of inhabitants above the age of 65 years was increasing faster than in other parts in Finland, and costs of delivering secondary care were high, especially for chronic conditions, such as heart failure, coronary heart disease and diabetes. The health-coaching call centre was established in the city of Lahti as a public–private partnership, where the public partner was responsible for the primary care and secondary care in the region.

The objective of this study was to evaluate the cost effectiveness of 12 months of telephone-based health-coaching intervention (the TERVA trial) for chronically ill patients in Finland. This was tested using a two arm trial with three patient groups with sub optimally controlled disease: type 2 diabetes mellitus (T2D), coronary artery disease (CAD) or congestive heart failure (CHF). The primary outcomes of the TERVA trial, the short-term clinical outcomes at 12 months follow-up, have been reported earlier [15].

## Methods

The total population of the area involved the health coaching program was approximately 112 000. Patients were recruited from electronic patient laboratory records in secondary care according to laboratory inclusion criteria (Glycated Hemoglobin (HbA1c) >7 or total cholesterol >4,5 or low density lipoprotein (LDL) >2.3 previous six months). In this phase we identified about 5 500 patients. After that research nurse identified those patients who were applicable for coaching according to inclusion and exclusion criteria from patients’ medical records, 2594 patient fulfilled inclusion criteria and were invited to participate. The information and consent letters were sent to the patients. 1535 identified patients, gave consent and were randomized to either control (C) or intervention (I) groups. At the baseline, there were no significant differences in age, sex, self- reported duration of disease and age of diagnosed, blood pressure (systolic, diastolic), total cholesterol, high density lipoprotein (HDL), LDL, body mass index (BMI), waist circumference, daily smokers, lipid lowering medication, HbA1c, oral antidiabetic drug and insulin, oral antidiabetic drug, insulin and NYHA-class between intervention and control groups [15]. Randomization 2:1 ratio was intentional for practical reasons. Statistical power calculations were conducted to verify that the imbalance would not cause problems. The intervention group received monthly individual health coaching for 12 months in addition to routine social and healthcare. Patients with multiple morbidities received coaching for each disease according to their personal priorities. Patients in the control group received routine social and health care e.g. visited diabetes nurse and doctors in primary and secondary care. Patients with more than one disease were allocated to following hierarchy: CHF- CAD- T2D [15]. Of these 1535 participants, 998 patients with complete baseline and follow-up data were included in the cost-effectiveness analysis (83 patients in the CHF group (I 56, C 27), 192 in the CAD group (I 124, C 68) and 723 in the T2D group (I 505, C 218). A total of 537 patients were lost in the follow-up. The detailed recruitment and randomization process has been published previously [15].

### Intervention

Eight experienced certified nurses and public health nurses were hired and trained in the motivational interviewing technique and coaching by telephone. Health coaches had access and the possibility to document patient health status into the primary and secondary care electronic health records (EHR), but they were not integrated in the care teams in the primary care centres. A more complete description of the health-coaching intervention process can be found in [15].

The health-coaching intervention included eight key recommendations developed by Pfizer Health Solution (PHS) and were adjusted for the Finnish healthcare system and Finnish evidence-based guidelines. The eight recommendations included: 1) know how and when to call for help, 2) learn about the condition and set goals, 3) take medicines correctly, 4) get recommended tests and services, 5) act to keep the condition well, 6) make lifestyle changes and reduce risk, 7) build on strengths and overcome obstacles and 8) follow up with specialists and appointments. Coaching was technology supported and utilized a traffic-light system for patients’ progress in relation to the key recommendations. Patient’s self-management booklets supported progress towards the key recommendations. Each disease had a separate booklet prepared in collaboration with the Finnish Heart Association and the Diabetes Association. The patients in the intervention group were called approximately 10–12 times during the intervention period.

### Data

#### Health-related quality of life

HRQoL was measured by using 15D [16].15D is a generic, self-administered instrument for measuring HRQoL among adults (age over 16 years) with 15 dimensions: mobility, vision, hearing, breathing, sleeping, eating, speech, excretion, usual activities, mental function, discomfort and symptoms, depression, distress, vitality and sexual activity. Completing the questionnaire takes 5–10 min. Each dimension has five ordinal levels, and 15D can be used as a profile measure or a single index number on a scale of 0–1 (0 dead, 1 completely healthy). Typically, 15D is used to measure the effectiveness of a single intervention [16, 17] and performs well in comparison to SF-36 as a HRQoL-instrument [18].

The baseline HRQoL data were collected by sending the 15D questionnaire to patients in the intervention and control groups at the beginning of the health-coaching intervention and follow-up measurements were made when the coaching finished after 12 months.

#### Cost data

Data for the costs and use of social and healthcare services were collected from the National registries maintained by the National Institute for Health and Welfare (Dnro THL/119/5.05.00/2015). These registers included the hospital benchmarking database the National Discharge Registry (HILMO) and Care Registers for Social Welfare (SosiaaliHILMO). Using a unique patient identification code, patient cohorts were linked to the registers, and all use of social and healthcare during 1-year follow-up was included for each individual. Secondary care data included the use of hospital outpatient care (all types of visits) and hospital admissions (diagnosis-related groups [DRGs]). Social care data included all types of long- and short-term institutionalized care, housing and residential services and home care services.

Hospitalizations and hospital outpatient visits due to any cause were extracted from the Hospital Discharge Register based on the International Classification of Diseases 10th revision (ICD-10) codes, the Finnish version of the Nordic Classification of Surgical Procedures (NCSP) codes for diagnostic and treatment procedures and the respective NordDRG patient grouping classifications. The DRG cost weights for hospitalizations and outpatient visits were based on individual-level cost-accounting data from several hospitals. The unit cost estimates for social care encounters and bed days were derived from the national price list for unit costs of healthcare services in Finland [19].

The use of primary healthcare resources was collected directly from the patient administration system (PAS) containing patient-level data abstracts from the electronic patient records. The PAS data included contact types (such as a visit, phone call or electronic messaging), patient’s age, the diagnosis (ICD-10) or the reason for encounter (ICPC-2) and the employee category of the healthcare professional in the contact. Extracting the patient-level data from the patient administration systems (with diagnosis and activity information) made it possible to group each individual encounter type by the Ambulatory and Primary Care Related Patient Groups (APR) grouper software, a grouping system equivalent to DRGs used in hospital care [20]. The batch grouper software assigned each individual patient encounter in one of the 44 APR groups. After grouping, each of the 44 APR groups in the sample was assigned a cost weight indicating the relative consumption of resources. Cost weights were based on large samples of time measurements in primary care contacts and procedures. All costs were deflated using the price index for public healthcare provided by Statistics Finland.

#### Statistical analysis

We report differences in the mean costs and outcomes and the corresponding cost-effectiveness ratio (ICER). ICER is defined by the difference in cost between the intervention and control, divided by the difference in their effect.

Uncertainty in the ICER estimates was accounted for by generating bootstrap 1000 replicates of the dataset, a method widely used in health economic evaluations [21, 22] to study the likelihood of effectiveness of an intervention in relation to the costs of care induced by the intervention [23]. Probabilistic sensitivity analysis was completed by calculating the cost-effectiveness acceptability curve (CEAC) derived from the bootstrap replicates. CEAC indicates the probability for cost effectiveness of the intervention at different levels of willingness to pay for the additional health outcome [24].

## Results

The overall incremental ICER was €48,000 per QALY. The cost effectiveness of health coaching was highest in the T2D group (ICER €20,000 per QALY). The ICER for the CAD group was more modest (€40,278 per QALY), although the improvement in QoL was greatest in this group and also exceeded the threshold for a clinically significant change in 15D (>0.015 [25]). In the CHF group, the effect on QoL was slightly negative at an increased cost (Table 1).

**Table 1:** Incremental costs, quality of life and cost-effectiveness ratios in the disease subgroups and in the whole study group

	Costs (€), mean (95% CI)			QoL (15D), mean (95% CI)			ICER (€/QALY)
	Intervention	Control	Incremental cost	Intervention	Control	Incremental effect	
Type 2 diabetes	1948 (1673–2222)	1788 (1204–2371)	160 (−406–726)	0.008 (0.003– 0.014)	0.000 (−0.009–0.009)	0.008 (−0.002–0.018)	20 000
Coronary artery disease	2510 (1806–3214)	1785 (984–2585)	725 (−389–1839)	0.019 (0.007–0.030)	0.001 (−0.014–0.016)	0.018 (−0.001–0.037)	40 278
Congestive heart failure	4469 (1955–6983)	2214 (−105–4533)	2255 (−1669–6180)	0.013 (−0.007–0.032)	0.015 (−0.015–0.046)	−0.003 (−0.037–0.032	-
All	2256 (1940–2571)	1824 (1345–2302)	432 (−135–999)	0.011 (0.006 − 0.015)	0.002 (−0.006–0.009)	0.009 (0.000–0.018)	48 000

*CI* confidence interval, *QoL* Quality of Life, *ICER* incremental cost-effectiveness ratio, *QALY* quality- adjusted life years

**Fig. 1:**
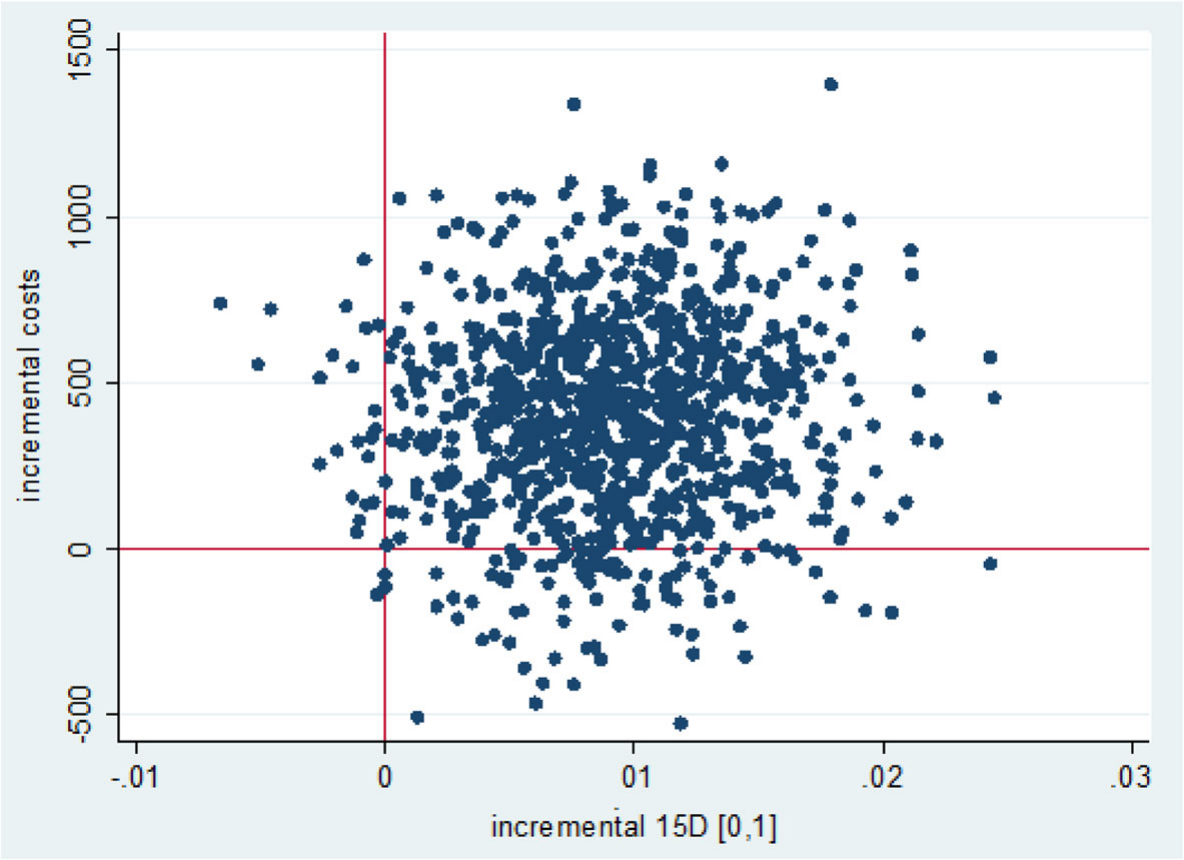
Distribution of bootstrapped incremental costs and health-related quality of life

Figure 1 presents the bootstrapped results among the whole study group displayed in a cost-effectiveness plane. There was considerable uncertainty in the ICER of the intervention.

The cost-effectiveness plane for HRQoL (15D) after health coaching showed that the intervention was more effective compared to care as usual but also more costly. Of the bootstrapped ICERs, 89% fell into the northeast quadrant, indicating increased QoL at an incremental cost; 9% of the points fell into the southeast quadrant, indicating increased QoL at a decreased cost. Only 2% of the data points fell into the northwest quadrant, and less than 1% fell into the southwest quadrant, suggesting a very small probability for a decrease in QoL at an incremental or decreased cost (Fig. 1).

**Fig. 2:**
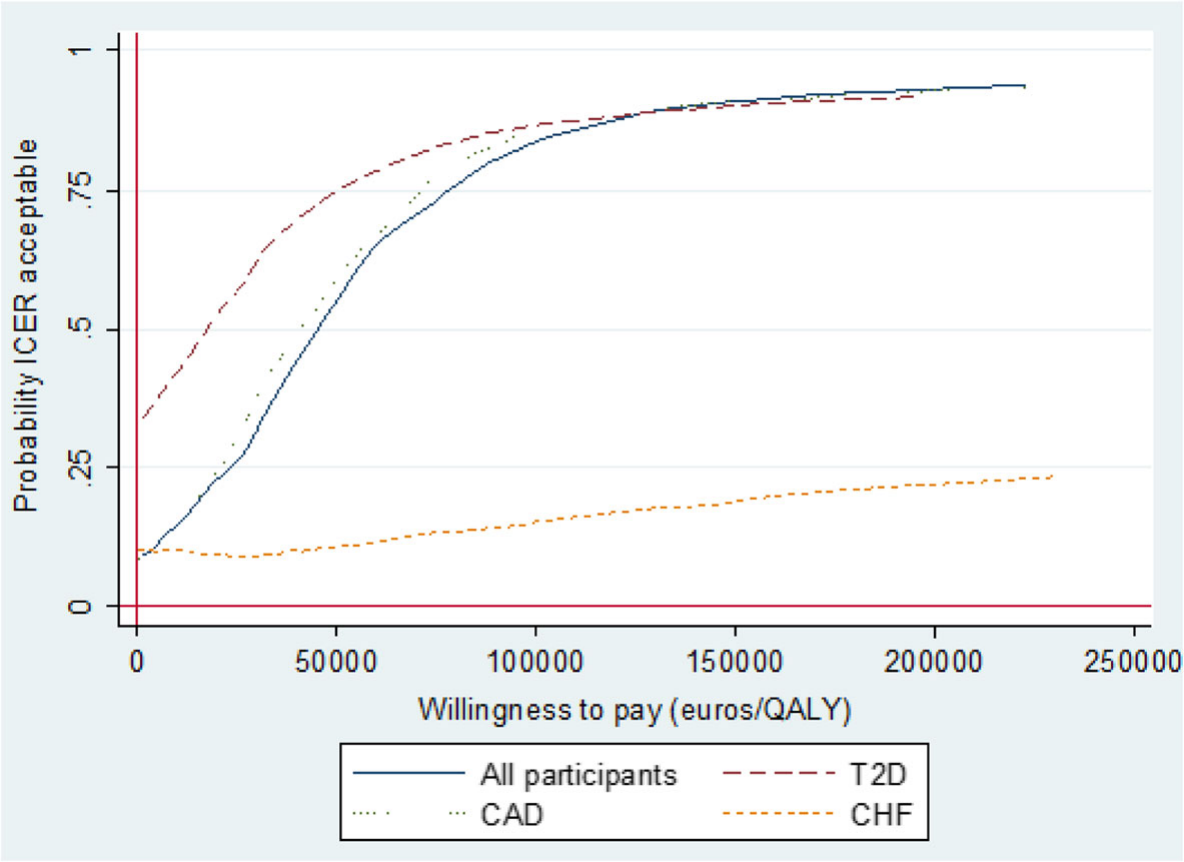
Cost-effectiveness acceptability curves for all participants and diagnosis-based subgroups

Figure 2 shows the incremental CEACs for the whole participant group and for the disease-specific subgroups. At no willingness to pay for incremental QALY, the probability of health-coaching cost effectiveness was less than 10% among all participants. At a willingness to pay €46,000 per QALY, the probability that the intervention is cost effective was over 50%. If the decision maker were willing to pay €50,000 per QALY, the probability of cost-effectiveness is 55%. The CEAC for the T2D group showed over 50% probability of cost effectiveness at a willingness to pay €20,000 per QALY. At a willingness to pay €50,000 per QALY, the probability that the intervention is cost effective for the T2D patients was 75%.

## Discussion

In this study, the cost effectiveness of 12 months telephone-based health-coaching intervention among three groups of chronically ill patients with unmet treatment goals was evaluated. The overall ICER was €48,000 per QALY. Further probabilistic sensitivity analysis showed a 55% probability of cost effectiveness if the decision maker were willing to pay €50,000 per QALY. Investments in programs for coaching patients may well be acceptable. Further disease-specific analyses indicated that the ICER for health coaching was lowest in the T2D group with a moderately low cost per QALY of €20,000. In the CAD group, the cost per QALY was higher (€40,278), and in the CHF group the effect on QoL was slightly negative at an increased cost.

Graves et al. [9] reported similar results ($29,375 per QALY, approximately €21,045 per QALY) for patients with T2D or hypertension after 1-year telephone-delivered intervention for physical activity and diet in a low socioeconomic area in Australia. Jacobs-van-der Bruggen et al. [26] analysed seven lifestyle interventions among patients with T2D and simulated the long-term outcomes. Health improvements were achieved at reasonable costs (≤ €50,000 per QALY), and average gained health-adjusted life years were 0.01–0.14 QALY per participant. Results in the CHF group somewhat contradicted the results of previous studies [5, 6]. However, the small number of patients (I 56, C 27) may have diluted the evidence in this subgroup or the coaching program did not support those people.

In this study, cost per QALY was found to be lowest in the T2D group. An improvement in QALY (0.008) was achieved with a small increase in the cost of care (€160 per patient). In the CAD group, both the improvements in QoL (0.018) as well as the increase in cost (€725 per patient) were higher. Possible explanations for the difference between these groups can be found in the medical history and the care received by the patients prior to the intervention. Most CAD patients were recruited for health coaching a few months after an acute percutaneous transluminal coronary angioplasty operation. Motivation to lifestyle changes and self-management are high after an acute cardiovascular attack [27]. The proximity of this severe incident may have activated the CAD patients in their self-care and healthcare service use and therefore fortified the effect of the intervention on the QoL increased cost in this group. Another reason for the increased cost in this group can be attributed to the standard follow-up visits after an acute cardiovascular attack or intervention in secondary care. Further, in care as usual, the diabetes patients receive treatment and self-care support from specially trained diabetes nurses, while the self-care support for CAD patients is not arranged as systematically in the present healthcare provision. This may explain the difference in the increased cost of care between the groups.

This study is among the few cost-effectiveness evaluations of health coaching for the chronically ill carried out in a real-life setting and using RCT design. Another strength of the present study was the use of national registries and local patient administration systems, including all social and healthcare services and their costs in the follow-up. Many studies published so far have relied on the self-reported use of services.

One clear shortcoming in the study was the rather short follow-up period. Significant health behaviour changes take at least 6 months and may have delayed the impact in clinical changes [28, 29]. A new, long term follow-up study, using the cohorts in the present study and based on National registries, has been set up to clarify the effects by analysing the differences in distal end points (such as complications in T2D and major events in CAD) and the differences in cumulated health and social care costs.

Immediately after the TERVA trial, only the clinical results and direct cost data were available for the regional decision makers, and the health-coaching program was cancelled. This may be a common problem with the evaluation of self-management and other preventive interventions, which typically focus on short-term health outcomes [30]. In this study, a closer exploration using QALYs and subgroup analysis revealed that closing the coaching program may have been questioned on the basis of the cost-effectiveness analysis.

We conclude that the assessment of cost effectiveness in preventive actions is demanding and thus requires careful and balanced analyses to sufficiently inform the decision makers on preferred choices.

## Conclusions

Decision makers in health care are actively seeking interventions leading to better health outcomes with a lower cost, but the evidence on cost effectiveness of self-management interventions is still scarce. In this RCT conducted in a real-life primary care setting, health coaching improved the QoL of T2D and CAD patients with moderate costs in the short-term follow-up. The results of our study suggest that health coaching should be targeted to selected patient groups. However, the follow-up period was probably too short to evaluate the cost-effectiveness of health-coaching intervention and a long-term evaluation is needed.

## Abbreviations

APR: Ambulatory and primary care related patient group
BMI: Body mass index
CAD: Coronary artery disease
CEAC: Cost-effectiveness acceptability curve
CHF: Congestive heart failure
CI: Confidence interval
DRG: Diagnosis-related groups
EU: European Union
EVO: Special state funding
HbA1c: Glycated hemoglobin
HDL: High density lipoprotein
HILMO: National discharge registry
HRQoL: Health related quality of life
ICD-10: Classification of disease 10^th^ revision
ICER: Incremental cost-effectiveness
ICPC-2: Reason for encounter patient administration system
LDL: Low density lipoprotein
NCSP: Nordic classification of Surgical Procedures
NYHA: New York heart association index on angina pectoris
PAS: Patient administration system
QALY: Quality adjusted life years
QoL: Quality of life
RCT: Randomized controlled trial
Sos. HILMO: Care register for social welfare
T2D: Type 2 diabetes
TERVA: Telephone-based health coaching intervention
